# Modification in Structures of Active Compounds in Anticancer Mitochondria-Targeted Therapy

**DOI:** 10.3390/ijms26031376

**Published:** 2025-02-06

**Authors:** Agnieszka Pyrczak-Felczykowska, Anna Herman-Antosiewicz

**Affiliations:** 1 Department of Physiology, Medical University of Gdańsk, 80-211 Gdańsk, Poland; 2 Department of Medical Biology and Genetics, Faculty of Biology, University of Gdańsk, 80-308 Gdańsk, Poland; anna.herman-antosiewicz@ug.edu.pl

**Keywords:** mitochondria, metabolism, cancer, natural compounds, structure modification, anticancer agents, synthetic derivatives, nanoparticles, conjugates, targeted therapy

## Abstract

Cancer is a multifaceted disease characterised by uncontrolled cellular proliferation and metastasis, resulting in significant global mortality. Current therapeutic strategies, including surgery, chemotherapy, and radiation therapy, face challenges such as systemic toxicity and tumour resistance. Recent advancements have shifted towards targeted therapies that act selectively on molecular structures within cancer cells, reducing off-target effects. Mitochondria have emerged as pivotal targets in this approach, given their roles in metabolic reprogramming, retrograde signalling, and oxidative stress, all of which drive the malignant phenotype. Targeting mitochondria offers a promising strategy to address these mechanisms at their origin. Synthetic derivatives of natural compounds hold particular promise in mitochondrial-targeted therapies. Innovations in drug design, including the use of conjugates and nanotechnology, focus on optimizing these compounds for mitochondrial specificity. Such advancements enhance therapeutic efficacy while minimizing systemic toxicity, presenting a significant step forward in modern anticancer strategies.

## 1. Introduction

Cancer is a diverse group of diseases characterised by uncontrolled proliferation of abnormal cells that invade nearby tissues and spread to other body parts. Such metastases are responsible for the majority of cancer patients’ deaths. Due to its prevalence, cancer therapy has become a substantial focus among healthcare providers and researchers. The Global Cancer Incidence, Mortality and Prevalence (GLOBOCAN) platform provides comprehensive cancer statistics, including incidence and mortality estimates for 36 cancer types across 185 countries. Data from 2020 indicate that, globally, one in five individuals will develop cancer, with mortality rates of one in eight for men and one in eleven for women. The rising prevalence of cancer can be attributed to an ageing population and various socio-economic risk factors. It is estimated that the number of cancer patients will rise to 22 million within the next twenty years [1]. The increasing global burden of cancer places a strain on individuals, families, communities, and healthcare systems, particularly in low- and middle-income countries lacking adequate resources for managing this challenge. 

Cancer research presents significant challenges due to the intricate nature of the disease. The genetic mutations, organ-specific manifestations, prognosis, and treatment strategies can vary greatly across different cancer types [2]. The success of available treatments, such as surgery, radiation therapy, chemotherapy, and immunotherapy, largely depends on the cancer’s type and stage at the time of diagnosis. While surgery and radiation therapy provide localised treatment, chemotherapy exerts systemic effects, often requiring a combined approach based on the specific cancer characteristics [3]. 

The primary drawback of chemotherapy is its lack of selectivity, which leads to significant toxicity and side effects by affecting both cancerous and normal cells. Another major challenge in cancer treatment is the development of resistance to chemotherapy, radiation therapy, and immunotherapy, which leads to tumour recurrence and metastasis [4]. This resistance accounts for 90% of cancer-related deaths, making it a critical focus in oncology [5,6]. However, in the past two decades, cancer treatment has shifted significantly from broad-spectrum cytotoxic drugs to targeted therapies [7,8]. 

Targeted therapy differs from chemotherapy in using compounds that target specific molecular structures (molecules or processes) within cancer cells. Unlike traditional chemotherapy, targeted drugs can specifically attack cancer cells while sparing normal cells, resulting in higher efficacy and lower toxicity [9,10]. Targets in this approach are believed to be genetically altered in cancer cells and are critical for tumour growth and survival and in this regard, mitochondria play a crucial role. The multifaceted roles of mitochondria lead to their involvement in cancer development. Mitochondria are implicated in the formation of the malignant phenotype in several ways concerning the metabolic reprogramming of cancer cells. DNA mutations affect signalling to the nucleus, triggering changes in cellular development and nuclear DNA replication (known as ’retrograde signalling’) [11]. They also influence the metabolic status of neighbouring stromal cells (referred to as the ’reverse Warburg effect’) [12,13,14]. These changes are linked to various cancer diseases, including hepatocellular carcinomas and prostate cancers [15]. In this context, mitochondria remain the crucial object in targeted anticancer therapy.

This review discusses why synthetic derivatives of natural compounds represent excellent candidates for designing modern anticancer therapies. Additionally, it highlights key aspects of selecting research models for testing potential anticancer compounds. The paper addresses molecular targets related to mitochondrial function, such as mitochondrial DNA (mtDNA), mitochondria-dependent reactive oxygen species (mtROS) generation, and their role in inducing regulated cell deaths, including apoptosis and ferroptosis. It also explores the interference of synthetic anticancer agents with mitochondrial metabolism, with a focus on the tricarboxylic acid (TCA) cycle, oxidative phosphorylation (OXPHOS), and electron transport chain (ETC). Lastly, it emphasises modern strategies for designing anticancer compounds targeting mitochondria.

## 2. Synthetic Derivatives of Natural Compounds as the Key Players in Drug Development

An analysis of new drug sources from 1981 to 2010 revealed that only 36% of new chemical entities were discovered without natural product inspiration [16]. A major challenge in discovering novel natural products is dereplication, which involves identifying and excluding already known substances. From 2001 to 2010, there has been a downward trend in the number of new chemical entities discovered annually. There is a hypothesis that industry relies heavily on combinatorial chemistry [17]. This is likely because the chemical structure of a naturally derived compound cannot be patented without prior modification. Computational techniques, like virtual screening, have not yet been effective in producing good lead compounds, indicating that drug discovery remains a highly complex process. Thus, natural products still serve as excellent starting points for the synthesis of analogues [18]. Additionally, the chemistry associated with natural compounds offers opportunities to explore new reactions and develop innovative synthetic strategies.

Nature provides a wealth of anticancer drugs due to its diverse chemotypes and pharmacologically active metabolites [19]. Despite the historical significance of natural products as a pivotal source of therapeutic agents, these compounds can occasionally exhibit suboptimal efficacy, unfavourable pharmacokinetic profiles, deleterious toxicity, or limited bioavailability, which constrain their direct clinical application. Only a small percentage of natural metabolites have been developed into clinically active drugs, but they serve as leads for the synthesis of more effective analogues and prodrugs. Modern techniques such as metabolomics, total or combinatorial synthesis, and biosynthetic pathway modification can be used to enhance the activity of these compounds [20].

The optimisation of natural lead structures focuses on enhancing drug efficacy, optimizing ADMET (Absorption, Distribution, Metabolism, Excretion, and Toxicity) profiles, and improving chemical accessibility (for further description see [21]). Traditionally, the primary goal has been to increase efficacy, using classic medicinal chemistry and modern rational drug design techniques. However, recent recognition of poor pharmacokinetic profiles and unacceptable toxicity as major causes of drug development failures has shifted the focus to optimizing ADME properties and reducing toxicity. For natural products, their structural complexity often negatively affects pharmacokinetic properties, necessitating careful optimisation of solubility, cellular permeability, and stability. Improving the chemical accessibility of natural leads is also crucial, as limited availability and synthetic challenges frequently hinder their development. Rational drug design efforts can overcome these challenges, facilitating the therapeutic application of natural product-based drugs [22].

In some cases, the extraction of natural bioactive compounds can meet difficulties, and total synthesis must be utilised. Importantly, during this process, one can identify the essential sub-structural portion of a molecule necessary for activity, leading to simpler analogues with similar or better activity than the natural product. One of the most spectacular examples is the marine-derived antitumor agent halichondrin B, where synthetic studies showed that the right half of the molecule retained most of its potency. This led to the development of the analogue E7389 (eribulin), which was approved by the FDA in 2010 as a third-line treatment option for metastatic breast cancer patients who had already undergone treatment with an anthracycline and a taxane [23].

Organisms provide complex libraries of unique bioactive constituents, similar to the libraries created by combinatorial chemistry. The natural products approach complements the synthetic approach, offering different initial lead structures. However, combinatorial chemistry may be a powerful tool for optimizing the structures of novel drugs. The task of researchers is to select pharmacologically interesting lead compounds from the libraries of extracts of natural origin [19].

## 3. Choosing an In Vitro Experimental Model for Anticancer Drug Development 

**Figure 1:**
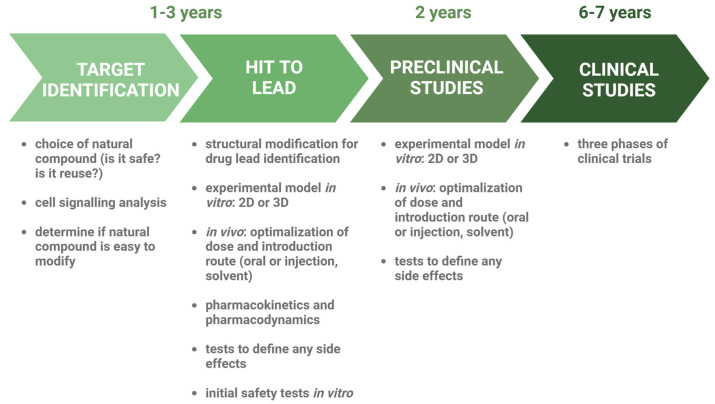
Development of novel anticancer drugs. Stages of drug creation are marked in green arrows. Descriptions of particular steps are provided below the arrows.

Typically, the development of anticancer drugs is a long process, which includes a preclinical phase followed by three clinical phases (Figure 1). Preclinical studies, primarily based on two-dimensional (2D) cell cultures and animal models, are essential in translational cancer research [24]. In 2D in vitro experiments, cell cultures are exposed to drug candidates at constant concentrations for a set period of time to screen and rank a large number of compounds based on their anticancer effects, typically measured by cell viability. These studies also assess other endpoints like target engagement and downstream effects to evaluate potency and mode of action. Compounds with promising efficacy in 2D systems advance to in vivo animal model tests. Despite their popularity due to easy handling, reproducibility, and low cost, 2D cell cultures cannot fully replicate the properties of in vivo solid tumours. Therefore, they are limited in their ability to predict the complex interactions and responses seen in actual tumours [25,26].

The abnormal vascularisation of solid tumours creates microenvironments deprived of oxygen and nutrients, where cells grow slowly or become quiescent, showing different phenotypic characteristics compared to those in well-vascularised regions [27]. These non-dividing cells often resist standard chemotherapies that target DNA replication and cell division [28]. Their altered phenotype allows them to survive chemotherapy and restart tumour growth after treatment ends. Therefore, cancer drug discovery needs to focus more on screening for agents that can target and eliminate quiescent, metabolically stressed cells [29].

An alternative strategy for cell-based screening for new anticancer drugs, which represent the characteristics of three-dimensional (3D) solid tumours more accurately, is the application of 3D cancer cell cultures [30]. The multicellular tumour spheroid model, which is more complex than monolayer cultures but less complex than in vivo tumours, is more suitable for drug screening and evaluation. Spheroids are more resistant to drugs due to pharmacokinetic obstacles limiting their penetration into 3D structure and multicellular interactions altering gene and protein expression [31,32]. Growing cells in 3D also allows for exploring vulnerabilities in the core regions related to hypoxia and nutrient deficiency, reflecting the heterogeneous tumour microenvironment. Poorly vascularised and perfused tumour micro-areas in aggressive cancers have limited access to oxygen and glucose, leading to metabolic changes and increased lactic acid production, resulting in an acidic pH in the tumour microenvironment [33]. This model provides a more accurate representation of the challenges faced in targeting tumours in vivo [29].

A significant limitation of 3D cultures is the requirement to dissociate single cells from spheroid structures through proteolytic degradation, a process that may take from several hours to a few days [26,34]. In comparison to 2D cultures, many 3D systems demonstrate reduced efficiency, shorter lifespan, lower repeatability, and decreased ease of use. Moreover, it is frequently highlighted that 3D structures often form not from individual cells but from clusters, which can impact experimental outcomes (for a review, see reference [35]). Anyway, these multicellular aggregates retain a three-dimensional architecture and are considered to be a more accurate model simulating in vivo conditions as compared to two-dimensional adherent cultures. 

While 3D culture systems are crucial for studying new anticancer drugs, their use in cell biology is not yet widely adopted. Most biological assays and imaging methods are optimised for 2D models, making the transition to 3D systems challenging. Recent advances in imaging techniques, such as multi-photon and light-sheet microscopy, have improved the quality of 3D imaging, but challenges like sample preparation and staining protocols still need to be addressed for broader implementation [36]. 

## 4. Mitochondria as Targets of Anticancer Therapies

Targeting mitochondria has garnered substantial interest in medicine since the 1950s when molecules with mitochondrial affinity were identified. Mitochondria play a crucial role in regulating various cellular functions, including ATP production, regulation of calcium levels and redox status, ROS generation, participation in biosynthetic pathways, and apoptosis initiation [37]. 

Cancer cells heavily rely on oxidative glycolysis, leading to increased levels of glucose transporters and a high glycolytic rate, which results in altered pH conditions favouring cell proliferation [38,39,40]. Thus, cancer cells undergo bioenergetic reprogramming, shifting from maximal ATP production via OXPHOS, seen in healthy cells, to generation of substrates for rapid cellular growth and division by glycolysis [15]. However, the metabolic needs and antioxidant defences in cancer cells surpass those of quiescent cells, making mitochondria vital for cancer cells’ proliferation [41,42,43]. Mitochondria play a crucial role in tumorigenesis through metabolic reprogramming, oxidative signalling, and ROS generation [44,45]. Moreover, distinct ion pumps and transporters in cancer cells contribute to changes in pH, aiding metastatic progression [46]. 

Mitochondria in cancer cells exhibit hyperpolarisation, potentially due to altered protein expression and increased intracellular Ca^2+^ levels [38,47]. This hyperpolarisation, alongside elevated ROS levels, contributes to enhanced cancer cell survival, migration, and invasion [46,48,49]. At the same time, the increased potential of the mitochondrial membrane results in preferential accumulation of mitochondria-targeting compounds in cancerous cells [50,51]. 

ROS, generated primarily by mitochondria, play a crucial role in cancer progression, acting as signalling agents or species toxic to cellular components [13,37]. mtROS production and redox balance are influenced by the electrochemical gradient across the inner membrane [52]. ATP synthase utilises this gradient to produce ATP, but its inhibition by oligomycin enhances mitochondrial membrane polarisation, leading to the superoxide generation [52,53]. In carcinomas, ATP synthase inhibition can occur due to increased levels of ATPase inhibitory factor 1 (IF1) [54] or hindrance in ATP synthase catalytic β-subunit transcription/translation [55,56]. Decreasing IF1 levels in carcinomas reduces glycolysis and enhances OXPHOS, akin to the effects of oligomycin, leading to increased mitochondrial membrane potential, ETC stalling, and higher ROS production [15,45,57].

Mitochondria also regulate cell death pathways such as apoptosis, ferroptosis and necroptosis, influencing cancer progression and treatment resistance. Mitochondrial function is heavily influenced by Ca^2+^ regulation [58]. Mitochondria and endoplasmic reticulum (ER) have a close connection by the dynamic platform named mitochondria-associated membranes (MAMs) which bring the IP3R Ca^2+^-releasing channels in ER in proximity to the mitochondrial Ca^2+^ uniporter [58,59]. Inactivation of MAMs functionality leads to AKT-mediated hyperphosphorylation of IP3R3 channels, reducing Ca^2+^ flux. Inactivation of MAMs residues might limit excessive Ca^2+^ uptake by mitochondria, potentially reducing apoptosis initiation, thereby promoting increased survival of cancer cells [60].

**Figure 2:**
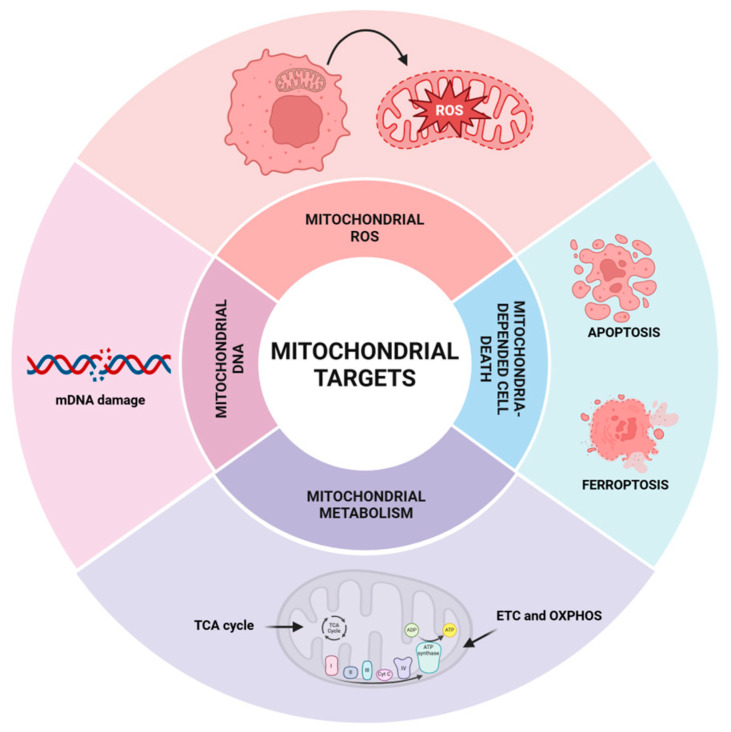
Mitochondrial targets in anticancer drug design.

**Figure 3:**
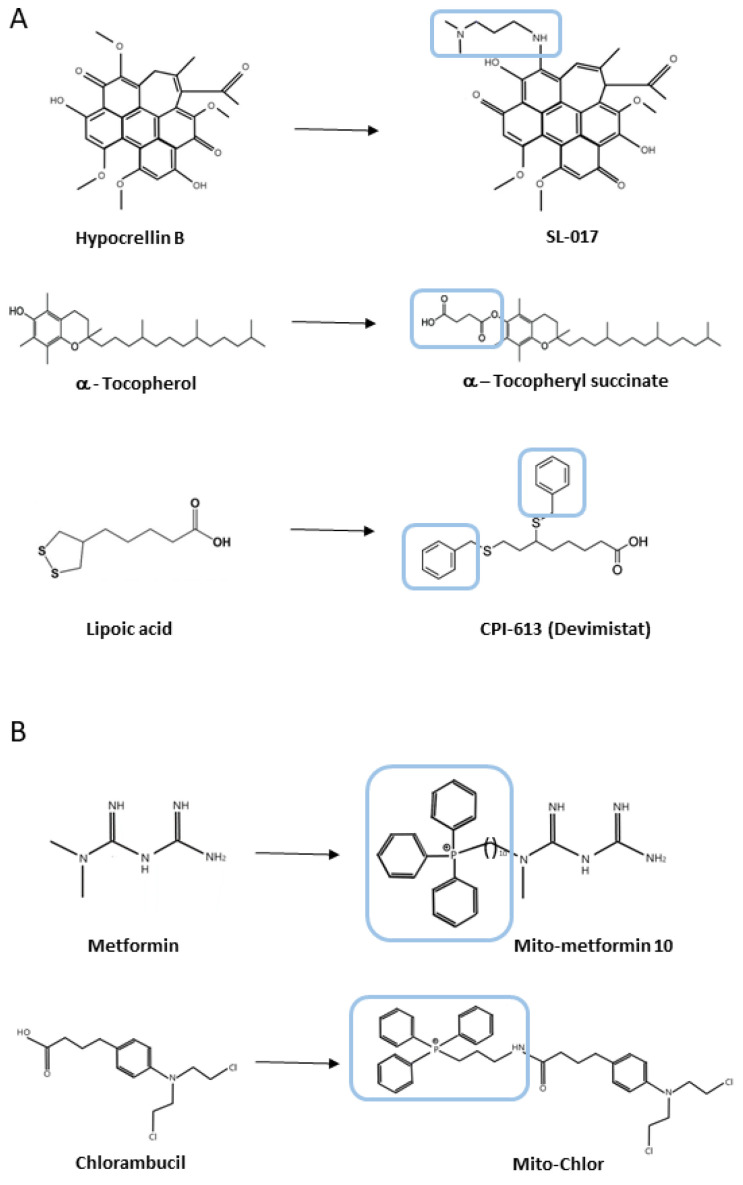
Examples of structural modifications of natural compounds in mitochondrial-targeted anticancer therapy. Direct modifications in structures (**A**) and conjugates with a TPP moiety (**B**) are presented.

Taking together, it is crucial to develop and utilize novel agents targeting mitochondria in cancer treatment. Main mitochondrial processes and molecules connected with targeted therapy in cancer cells are discussed below (see Figure 2), especially mitochondrial metabolism (Section 4.1) including the TCA cycle (Section 4.1.1), OXPHOS and ETC (Section 4.1.2), as well as mitochondrial ROS and cell death induction associated with oxidative stress (Section 4.2) and finally mtDNA (Section 4.3). The examples of synthetic analogues of natural bioactive compounds developed to target mitochondria functioning are presented in Table 1 and some of their structures in Figure 3.

### 4.1. Anticancer Agents Targeting Mitochondrial Metabolism

Mitochondria serve as essential bioenergetic centres where nearly all metabolic fuels are fully oxidised to CO_2_ and ATP via their conversion into acetyl coenzyme A (acetyl-CoA) and subsequent catabolic pathways. Key mitochondrial metabolic pathways include the TCA cycle, fatty acid oxidation (FAO), ETC, and OXPHOS, which collectively facilitate the catabolism of biomolecules and ATP production [61]. 

Advancements in understanding the deregulation of cancer metabolism have highlighted the potential of precision medicine to target specific tumour types. However, several challenges complicate metabolism-targeted therapy. Firstly, developing specific inhibitors is difficult due to the presence of multiple isoforms of many metabolic enzymes with high structural similarity, such as pyruvate kinase 1 and 2 [62]. Additionally, these enzymes often possess hydrophobic active site pockets that are challenging to target [63]. Furthermore, cancer cells can reprogram their metabolism, thus inhibiting one pathway by targeting a key enzyme might activate another metabolic route [64]. 

**Table 1:** Overview of compounds targeting mitochondria in cancer treatment.

Compound	Natural Compound Origin	Purpose of Synthesis	Type of Cancer	Delivery	Target	Study Advancement	Reference
SL017	hypocrellin B from *Hypocrella bambusae*	Increased biological activity Implementation in sonodynamic therapy	breast hepatic	Direct	Sonodynamic therapy (SDT) Generation of ROS Loss of mitochondrial membrane potential	Preclinical	[65]
Glycyrrhetinic acid derivative	glycyrrhetinic acid from *Glycyrrhiza glabra*	Improved delivery to mitochondria	breast ovarian colon cervical	TPP-conjugation	Mitochondrial membrane depolarisation after ROS generation	Preclinical	[66]
Amino acid esters of betulin	betulin	Increased solubility	epidermoid carcinoma	Direct	Decreasing the mitochondrial membrane potential	Preclinical	[67]
B63	curcumin	Increased biological activity and bioavailability	colon	Direct	Endoplasmic reticulum stress Mitochondrial dysfunction	Preclinical	[68]
CDDO-Me	triterpenoid	Increased biological activity	melanoma pancreatic renal	Direct	ROS generation Apoptosis induction Glutation level lowering	Clinical trial III phase– discontinued due to side effects	[69]
Mito-metformin_10_	guanidine	Improvement of mitochondrial targeting Increased biological activity	pancreatic	TPP-conjugation	Complex I inhibition	Preclinical	[70]
α-Tocopheryl succinate (α-TOS)	α-Tocopherol	Increased biological activity	breast	direct	Complex II inhibition	Preclinical	[71]
Compound 2a	curcumin	Increased biological activity	breast cervical hepatic	direct	Thioredoxin reductase inhibition	Preclinical	[72]
Synthetic α-methylene-δ-lactones	α-methylene-γ-lactones	Increased biological activity	breast	direct	Loss of mitochondrial membrane potential Change in Bax/Bcl-2 ratio Apoptosis induction	Preclinical	[73]
DOX-loaded pH-sensitive poly(β-amino ester)s polymers (PHP)-based micellar nanoparticles	doxorubicin	Overcome multidrug resistance	breast	pH-sensitive poly(β-amino ester)s polymers (PHP)-based micellar nanoparticles conjugated with doxorubicin	Lowering mitochondrial membrane potential and ATP level	Preclinical	[74]
DN3	jaridonin—a nature ent-kaurane diterpenoid extracted from *Isodon rubescens*	Increased biological activity Improved stability	gastric	direct	Mitochondrial membrane potential decrease and cytochrome c release	Preclinical	[75]
ABT-737	gossypol derivative	Increased biological activity	ovarian	direct	Mimicker of the Bcl-2 homology-3 (BH3) domain	Preclinical	[76]
CDDO derivative named 5b	pentacyclic triterpenoid oleanolic acid (OA) derivative	Increased biological activity and selectivity for the tumour cells	breast	TPP-conjugation	Mitochondrial membrane potential decline Mitochondria-mediated apoptosis	Preclinical	[77]
Atovaquone	ubiquinone	Increased biological activity	hypopharyngeal colorectal lung	direct	Mitochondrial Complex III inhibitor	Clinical trials to repurpose (actually used as antimalarial drug)	[78,79]
Compound 7	withangulatin A	Increased biological activity	breast (TNBC)	direct	Mitochondrial glutaminase (GLS1) inhibitor	Preclinical	[80]
CPI-613 (Devimistat)	lipoate	Natural ligand analogue	pancreatic lung	direct	KGDHC inhibitor	Clinical trials phase I and III	[81,82]
N-(4-hydroxyphenyl) retinamide (4HPR)	retinoic acid	Natural ligand analogue	ovarian prostate cervical	direct	Mitochondria-mediated apoptosis	Preclinical	[83,84,85]
Artesunate	artemisinin	Increased biological activity Improved water solubility	pancreatic liver ovarian breast lung	direct	GSH depletion Ferroptosis induction	Clinical trials to repurpose (actually used as antimalarial drug)	[51,86,87]
Alovudine	thymidine dideoxynucleoside	Increased biological activity	Acute myeloid leukemia (AML)	direct	Polymerase γ inhibitor Depletion of mtDNA Reduction in mitochondrial-encoded protein levels	Preclinical	[88]
Mito-Chlor	TPP derivative of chlorambucil	Increased biological activity Overcome the chlorambucil resistant	breast pancreatic	TPP conjugation	mtDNA damage Cell cycle arrest	Preclinical	[89]
Quinacrine	acridine derivative	Increased biological activity	oral squamous cell carcinoma	Gold NPs	ROS generation Mitochondria-mediated apoptosis	Preclinical	[90]
COUPY	curcumin derivative (fluorophor)	Photosentitizer—adaptation to PDT	cervical	direct	mtROS-dependent autophagy (PDT)	Preclinical	[91]

#### 4.1.1. Targeting the TCA Cycle

The TCA cycle is crucial for energy metabolism, macromolecule synthesis, and maintaining redox balance. This cycle consists of a series of biochemical reactions in the mitochondrial matrix, enabling aerobic organisms to oxidize fuel sources for energy and biosynthetic precursors [92]. Despite the prevalent view that cancer cells rely mainly on aerobic glycolysis, recent studies have highlighted the significant role of the TCA cycle in cancer metabolism and tumourigenesis [93]. This emerging understanding underscores the importance of the TCA cycle in the metabolic adaptation of cancer cells. Consequently, the TCA cycle’s contribution to cancer progression is gaining increasing recognition.

Research has shown that tumour cells can separate glycolysis from the TCA cycle, enabling the utilisation of alternative fuel sources like glutamine (process called glutaminolysis) to satisfy their increased metabolic demands [94]. Glutamine has been identified as a critical nutrient source in various cancer types, particularly those driven by MYC oncogene [95]. Inhibition of mitochondrial glutaminase (GLS1), the enzyme responsible for converting glutamine to glutamate, has shown effectiveness in mouse models of lung adenocarcinoma with Keap1 loss, renal cell carcinoma, and MYC-driven lymphoma [96,97,98]. Zhou et al. developed a series of semi-synthetic derivatives of natural GLS1 inhibitor, withangulatin A [80]. One of them, named 7, demonstrated effective therapeutic activity against triple-negative breast cancer (TNBC). In MDA-MB-231 cells, compound 7 reduced cellular glutamate levels by inhibiting the glutaminolysis pathway, subsequently inducing apoptosis through increased reactive oxygen species generation. Molecular docking studies revealed that compound 7 demonstrated a distinct binding pattern compared to withangulatin A within the allosteric binding pocket of GLS1 [80].

Another target for TCA-focused anticancer drug design is alpha-ketoglutarate- dehydrogenase complex (KGDHC), a rate-limiting enzyme in the TCA cycle, which comprises three components: α-KG dehydrogenase (OGDH), dihydrolipoamide S-succinyltransferase (DLST), and dihydrolipoamide dehydrogenase (DLD) [92]. In colorectal cancer, OGDH is downregulated due to promoter hypermethylation, a phenomenon also observed in breast, lung, esophageal, cervical, and pancreatic cancers [99,100]. Interestingly, an alternative splice variant of OGDH, which is tumour-specific, is overexpressed in colorectal cancer [101]. CPI-613, a lipoate analogue, inhibits KGDHC, as lipoate serves as a cofactor for this enzyme complex. It induces mtROS by targeting DLD and suppressing DLST, the E2 subunit of KGDHC [102]. CPI-613 is currently undergoing clinical trials, either alone or combined with standard chemotherapy for cancer treatment [81,82] (Figure 3).

#### 4.1.2. Targeting the OXPHOS and ETC

ETC consists of four protein complexes (I-IV) that facilitate redox reactions, generating an electrochemical gradient. This gradient drives ATP production within the comprehensive system of OXPHOS. NADH and FADH2 function as electron donors, with oxygen serving as the terminal electron acceptor in the electron transport chain. Protons are transferred from the mitochondrial matrix to the intermembrane space by Complexes I, III, and IV, establishing a proton gradient. This gradient drives Complex V (ATP synthase) to channel protons back into the mitochondrial matrix and facilitate the synthesis of ATP [103,104]. 

Functional ETC is essential for tumour growth. As mentioned above, the centres of many solid tumours are poorly vascularised, leading to nutrient-poor environments with limited glucose and oxygen availability [105,106]. Despite these conditions, tumour cores maintain respiration because the electron transport chain can function optimally even at oxygen levels as low as 0.5% [107]. Consequently, poorly vascularised tumour cores, although limited in glucose, have sufficient oxygen to continue generating mitochondrial ATP for cell survival. Additionally, reducing ETC function impairs the oxidative TCA cycle, thereby inhibiting macromolecule synthesis needed for tumour growth [106]. 

Ashton et al. [78] demonstrated that the mitochondrial complex III inhibitor, atovaquone (lipophilic ubiquinone analogue), reduced tumour hypoxia in both human xenografts and cancer patients by lowering oxygen consumption, thereby increasing oxygen availability in tumours. In the other study, it has been shown that atovaquone alleviated hypoxia and synergised with the immune checkpoint blockade (ICB) antibody, anti-PD-L1, significantly enhancing tumour eradication rates in the syngeneic CT26 colorectal cancer model. This synergistic effect depended on CD8+ T cells, established a tumour-specific memory of immune response, and was not associated with toxicity [79].

Another ETC inhibitor, better known as a medication for type II diabetes, is metformin. Metformin is primarily recognised as a first-line treatment for type 2 diabetes due to its ability to reduce hepatic gluconeogenesis and enhance insulin sensitivity. Epidemiological studies have linked metformin use with a reduced incidence of cancer, suggesting its potential as an anticancer agent (for a review see [108]). Metformin uniquely enters mitochondria in a membrane potential-dependent manner, and once inside, it inhibits complex I, reducing the mitochondrial membrane potential and consequently decreasing further metformin import, thus preventing toxicity while providing therapeutic effects [109,110]. Further laboratory-based studies corroborate metformin’s anticancer properties, demonstrating its efficacy in reducing tumour growth [111,112]. Clinical trials have shown mixed results, with some reporting significant benefits in cancer patients, such as improved survival rates in lung adenocarcinoma patients treated with the combination of metformin with tyrosine kinase inhibitors [113]. 

### 4.2. The mtROS and Programmed Cell Death in Anticancer Treatment

ROS are highly reactive molecules derived from oxygen (O_2_) and produced within cells with high metabolic activity. Elevated ROS levels cause cellular damage, while moderate levels have signalling roles. Mitochondria generate mtROS, particularly through ETC complexes I and III, which reduce O_2_ to superoxide (O_2_•^−^), subsequently converted to hydrogen peroxide (H_2_O_2_) by superoxide dismutases (SODs) [114]. Cancer cells tend to have higher ROS levels than non-transformed cells due to oncogene activity, loss of tumour suppressor genes, metabolic deregulation, mitochondrial dysfunction, inflammatory responses, or genotoxic stress [115]. This increased ROS production is counterbalanced by a robust and tightly regulated antioxidant system, with mitochondria having their own set of antioxidant enzymes, such as glutathione reductases, catalases, peroxidases, and other NADPH-generating sources, distinct from the cytosolic antioxidant machinery [116]. 

The abnormal metabolism of cancer cells is geared towards sustaining their growth in challenging environments, such as low oxygen and nutrient availability. To continuously fulfil their heightened energy and biosynthesis demands, cancer cells must independently expand their metabolic pathways [117,118]. This heightened metabolic activity leads to an accumulation of metabolites, disrupting the balance between redox homeostasis and oxidative stress [119]. Persistent oxidative stress results in a significant build-up of ROS within the cancer cells. Elevated ROS levels that are not lethal can facilitate cancer cell proliferation, metastasis, and angiogenesis. However, when ROS levels become excessively high, surpassing the cells’ tolerance, they induce cell death, thereby exerting an anti-tumour effect [117,120].

Excessive ROS within cells can damage proteins, nucleic acids, lipids, membranes, and organelles, leading to cell death processes such as apoptosis. Mitochondria play a pivotal role in initiating apoptosis and act as both a source and target of ROS [116,121]. Elevated mtmtROS levels can trigger intrinsic apoptosis by releasing cytochrome c from the mitochondrial intermembrane space into the cytosol [122]. This chain of events consequently leads to activation of Caspase-9, and finally, the effector caspases such as caspase-3, -6, and -7, resulting in the cleavage of cellular proteins and cell death [117,123]. Importantly, ROS are also linked to the extrinsic apoptosis pathway by activating transmembrane death receptors like Fas, TRAIL-R1/2, and TNFR1. Activation of these receptors recruits adaptor proteins, such as FADD, and procaspase-8 or -10, forming death-inducing signalling complexes (DISCs), which activates caspase-8 and -10, leading to effector caspase activation and apoptosis [122,123,124].

Although pro-oxidative compounds have become very promising anticancer agents, they also produce ROS in normal cells, leading to a narrow therapeutic window and significant toxic side effects, which considerably restrict their clinical use. Lately, Liu et al. overcame this obstacle by developing derivatives that generate ROS selectively in cancer cells over in normal cells [125]. They synthesised a novel curcumin 5-carbon mono-carbonyl analogue with a piperidone linker, and the introduction of electron-withdrawing trifluoromethyl and electron-donating methoxyl groups at meta position on the aromatic rings relative to the linker. Curcumin piperine-linked derivative, named 2c, was identified as an inhibitor of thioredoxin reductase (TrxR). The thioredoxin system, comprising thioredoxin reductase (TrxR), thioredoxin (Trx), and NADPH, plays a critical role in maintaining cellular redox balance by reducing Trx disulfides and mediating redox signalling to prevent oxidative damage from ROS. Due to elevated oxidative stress, cancer cells often overexpress TrxR, making it a promising target for cancer-specific therapies. Thus, as 2c inhibited TrxR, it served as a selective ROS-generating agent in cancer cells. It exhibited a strong ability to selectively kill human non-small cell lung cancer NCI-H460 cells over normal human lung MRC-5 cells, outperforming conventional chemotherapeutic agents such as 5-fluorouracil and camptothecin [125].

Ferroptosis is a quite recently identified regulated cell death mechanism, marked by iron-dependent accumulation of ROS and lipid peroxidation products within cells. Biochemically, morphologically, and genetically, ferroptosis is distinct from other forms of regulated cell death. During ferroptosis, cells exhibit a rounded morphology, detachment, and reduced mitochondrial size, with increased mitochondrial membrane density, loss of mitochondrial cristae, and rupture of the outer mitochondrial membrane, resembling apoptosis. However, unlike apoptosis, ferroptosis does not involve changes in nuclear size or chromatin condensation [126,127,128].

Although ferroptosis-like cell death has been observed over the past few decades, it was only recently that Dixon et al. formally named this nonapoptotic form of cell death "ferroptosis." They identified that the oncogenic RAS-selective lethal compound erastin activated a unique lethal pathway, distinct from other well-known regulated cell death processes [126]. In mammalian cells, ferroptosis is primarily regulated by iron homeostasis, lipid metabolism, and glutathione-dependent redox balance. Recent advances in ferroptosis research have led to significant efforts to identify potent and druggable modulators of this cell death pathway for clinical use [129,130,131]. These efforts have opened new possibilities for developing innovative treatment strategies aimed at ferroptosis-related diseases, including cancer [132].

The sesquiterpene lactone, artemisinin, derived from *Artemisia annua* and traditionally used in Chinese medicine for over two millennia, is FDA-approved as an antimalarial drug, along with its more water-soluble derivative, artesunate. Over the past two decades, extensive pharmacological research has been conducted on artemisinin and its derivatives, with several phase I/II trials demonstrating significant antitumour efficacy and generally low toxicity [133]. These studies emphasize the importance of the endoperoxide (1,2,4-trioxane) structure as a key pharmacophore. The mechanism of action of artemisinin is based on the endoplasmic reticulum stress and ROS accumulation which lead to non-apoptotic cell death. Clinical studies indicate that artemisinin and its derivatives induce ferroptosis in cancer cells and can be used for treating various tumours, including liver, ovarian, breast, non-small cell lung, colorectal, cervical intraepithelial cancers, and retinoblastoma [51,134,135,136]. A phase I trial found that artesunate had mild, self-limiting side effects and increased clearance rates over time in breast cancer patients [137]. Artemisinin and its derivatives may improve tumour treatment outcomes by inhibiting angiogenesis and enhancing immunity in patients with primary liver cancer and haematological diseases [51]. Importantly, combining artesunate with anti-tumour therapies has proven safe and effective in ovarian cancer and non-small cell lung cancer, increasing sensitivity to cisplatin in ovarian cancer and improving disease control rates when used with NP (a chemotherapy regimen of vinorelbine and cisplatin) and sequential administration [138].

### 4.3. mtDNA

The mtDNA, consisting of 37 coding and essential RNAs genes, is critical for mitochondrial function, regulating metabolic activity and cellular processes [139]. Mutations and changes in copy number of mtDNA have been strongly associated with accelerated tumour growth, underscoring its significance in cancer development [140,141]. Compared to nuclear DNA (nDNA), mtDNA’s absence of introns, lack of histone protection, and limited DNA repair capacity make it more susceptible to damage, resulting in various types of lesions including single-strand breaks, abasic sites, oxidative base damage, and DNA crosslinking [142,143]. Endogenous factors like oxidative stress [144,145], enzyme-induced damage [146], and the absence of repair mechanisms, along with exogenous factors such as industrial byproducts, therapeutic drugs, and radiation, can induce mutations in mtDNA [147]. 

Recently, due to its crucial involvement in cancer development, therapies targeting mtDNA have gained significant attention as promising strategies for cancer treatment. Innovations such as immunotherapy inducing mtDNA release, therapy with mitochondria-targeted complexes, and combinations of chemotherapy and photodynamic therapy (PDT) have significantly advanced the field of anticancer treatments targeting mtDNA [142]. These approaches hold great potential in improving cancer treatment outcomes.

Lin and colleagues proposed the classification where therapeutic strategies targeting mtDNA are categorised into two groups: indirect and direct targeting methods [142]. Direct methods involve the use of molecules that specifically bind to or cleave mtDNA, aiming to achieve therapeutic effects through direct intervention. In contrast, indirect approaches focus on disrupting pathways crucial for mtDNA stability, leading to its damage and dysfunction [142,148]. These classification schemes help delineate the diverse mechanisms utilised in targeting mtDNA for therapeutic purposes.

When designing the direct approaches, preventing the unintended impact on nuclear genes while targeting mtDNA has posed a persistent challenge. To overcome this hurdle, the utilisation of nano-carrier systems for precise delivery of agents targeting mtDNA has garnered significant attention in recent years [149,150]. Various delocalised lipophilic cations, including triphenylphosphonium (TPP), rhodamine 123, dequalinium (DQA), guanidine, and F16, serve as mitochondria-targeting ligands [151,152]. These ligands can be chemically linked to various anticancer agents using covalent bonds, such as amide, disulfide, ester, ether, and hydrazine bonds [153]. The most widely applied nano-carriers in mitochondria-targeted therapy, including lipophilic cations and nanoparticles, are discussed in the following sections. 

Phototherapy represents a novel approach in cancer treatment that utilises light to activate drugs or materials aimed at disrupting mtDNA. Techniques such as PDT, photothermal therapy (PTT), and photochemotherapy (PCT) have demonstrated potential in recent research [142,154,155]. Metal complexes and diamond-like carbon (DLC) coating systems are frequently involved in these methods due to their distinctive optical and chemical characteristics.

The PDT utilises photosensitizers that generate ROS upon light exposure, causing oxidative damage to cancer cells. PDT offers several advantages, including targeted light-controlled treatment, minimal invasiveness, low drug resistance, and reduced side effects [156,157]. Among photosensitizers widely applied in PDT, one can distinguish 5-aminolevulinic acid (5-ALA), a precursor in the heme biosynthetic pathway, and its derivative, hexyl aminolevulinate (HAL). 5-ALA has been effectively utilised in intraoperative imaging of brain tumours due to its selective accumulation in tumour cells and fluorescence properties, allowing neurosurgeons to achieve more precise tumour resections [158,159]. HAL, on the other hand, has shown promising results in the treatment of bladder cancer and non-aggressive basal cell carcinomas [160]. 

Due to its administration prior to therapy, ALA can accumulate in malignant brain cells and infiltrate neoplastic regions beyond the tumour margins, a process facilitated by increased vascular permeability [161]. Within mitochondria, this compound is converted into Protoporphyrin IX (PpIX), which is further processed into heme via the enzyme ferrochelatase. Reduced expression of ferrochelatase in the affected areas may promote PpIX accumulation in brain tumours, contributing to its selective localisation within the tumour [162]. The FDA approval of 5-ALA for fluorescence-guided tumour resection has reignited interest in using PDT as a treatment option for glioblastoma multiforme (GBM) [163]. Hexyl ester of 5-ALA (HAL) strikes a balance between hydrophilicity and lipophilicity compared to other derivatives of ALA. Administering HAL intravesically enhances PpIX synthesis for 1 to 2 hours, achieving this with a 20-fold lower concentration of HAL [164].

Indirect targeted therapy encompasses three main categories: affecting the genetic functions of mtDNA, stimulating immune responses, and elevating ROS levels [165,166]. Disruption of these processes leads to mtDNA damage, ultimately inducing tumour suppression through mitochondrial dysfunction-mediated pathways. This approach highlights the intricate interplay between mitochondrial biology and cancer progression [146].

The replication and maintenance of mtDNA rely on DNA polymerase γ, which is essential for mtDNA replication and repair processes [167]. Consequently, DNA polymerase γ may serve as a potential target for the development of anticancer therapies. Acute myeloid leukemia (AML) cells and cancer stem cells exhibit an elevated dependence on OXPHOS, prompting an investigation into the effects of polymerase γ inhibitors in AML. One of them is alovudine, a thymidine dideoxynucleoside analogue developed by Yehudai and colleagues in 2019 [88] In AML cells, alovudine led to the depletion of mtDNA, a reduction in mitochondrial-encoded protein levels, and a decrease in basal oxygen consumption, resulting in impaired cell proliferation and reduced viability.

Although significant advances have been achieved in the identification of therapeutic targets and the design and production of anticancer agents, drug delivery methods and targeting still need to be improved. The next sections are devoted to novel strategies in cancer drug development, including drug carriers (Section 5) and nanotechnology (Section 6).

## 5. Modern Modifications in Structures of Natural Compounds Targeting Mitochondria

### 5.1. Conjugates

The advancement of therapeutic agents that specifically target mitochondria has gained considerable attention in cancer treatment. However, the intricate structure of mitochondria presents significant obstacles for many molecules attempting to reach their interior. The dual-layered membrane, consisting of a porous outer membrane and a protein-dense inner membrane, creates a barrier that complicates their entry into the organelle [168]. Molecules intending to traverse the membrane must overcome the activation energy associated with removing water molecules. Delocalised positive charge in molecules can lower this activation energy, facilitating efficient penetration into the mitochondria. 

Delocalised lipophilic cations (DLCs) serve as popular mitochondrion-targeted agents, facilitating the construction of molecule-based nano-carriers that selectively accumulate in mitochondria [169]. Delocalised lipophilic cations, characterised by their lipophilicity and positive charge, facilitate their diffusion across phospholipid membranes, including plasma and mitochondrial outer membranes. The delocalised positive charge enables these molecules to penetrate the highly negatively charged mitochondrial matrix, which result from a mitochondrial inner membrane potential (approximately −180 mV) generated by proton gradient [170,171]. The hyperpolarised mitochondrial inner membrane of cancer cells (−220 mV), compared to healthy mitochondria, enables the selective entry of positively charged molecules [37,172]. 

One of such cations is triphenylphosphonium ion (TPP), known for its mitochondria-targeting properties. TPP’s single positive charge delocalised over three phenyl groups, along with its hydrophobicity, promotes interaction with the inner mitochondrial membrane [173]. Driven by the membrane potential, TPP accumulates significantly within mitochondria, providing an effective targeting system. TPP-based systems offer stability in biological systems, and combination of lipophilic and hydrophilic properties, low reactivity toward cellular components, easy synthesis, and purification make TPP-based systems promising for targeting mitochondria in cancer therapy [152]. The mitochondria-targeting characteristic of the TPP moiety has been utilised to deliver various cargos to mitochondria, including antioxidants (MitoQ, MitoVitE, and MitoTEMPOL) [174,175,176] and anticancer agents (MitoMetformin, Mitoporphyrin, and TP187) [173,177,178] as well as probes for detecting ROS (MitoSox and MitoPyl) [179,180] and for mitochondrial imaging (NPA-TPP) [50]. 

A number of examples indicated the successful utilisation of TPP as a mitochondria-targeting ligand. For instance, vitamin E analogues, such as a-tocopheryl succinate (a-TOS) and vitamin E succinate (VES), were linked to the TPP moiety, and among them, MitoVES with an 11-carbon linker strongly induced apoptosis in cancer cells, showing minimal activity in non-malignant cells. MitoVES also demonstrated mitochondrial localisation, generating ROS and inducing mitochondria-dependent apoptosis in cancer cells [181]. Furthermore, Millard and colleagues compared the potency of different cationic moiety-linked chlorambucil conjugates in cancer cell lines [182]. Among these, a TPP-chlorambucil named Mito-Chlor exhibited 80-fold higher cytotoxicity and extensive accumulation in mitochondria, causing mtDNA damage and cell death across various breast and pancreatic cancer cell lines that show resistance to the parent compound. Unfortunately, it could not completely suppress tumour growth in vivo. AbuEid et al. (2022) demonstrated that TPP conjugated to trifluoromethyl, and methoxy mito-metformin (MMe) analogues showed enhanced selectivity toward cancer cells compared to MMe, while retaining the same signalling mechanism [183]. Similar results were obtained in the combination of other established drugs like doxorubicin, cisplatin, chlorambucil, and camptothecin with a TPP cation. Furthermore, in most cases, incorporating trifluoromethyl groups into TPP resulted in decreased toxicity in vivo while maintaining anti-tumour effectiveness, paving the way for safer development of these next-generation TPP-conjugated compounds [37,182,184,185].

The extensive use of TPP as the preferred carrier for mitochondrial targeting was initially based on the assumption that the TPP moiety was inert. However, recent studies have revealed that the TPP moiety adversely affects mitochondrial bioenergetics. TPP–linker conjugates, even without cargo, have been found to increase proton leakage and uncouple OXPHOS, resulting in reduced ATP generation efficiency [186,187]. Hypotheses suggesting the mechanism behind uncoupling induced by alkyl-TPP conjugates include the potential disruption of inner mitochondrial membrane integrity at elevated concentrations, resulting in proton leakage [188], or the possibility of ionic interactions with natural fatty acid anions to enhance their transfer across the inner mitochondrial membrane, functioning as proton carriers [189]. Structural modifications to TPP conjugates have primarily aimed at adjusting the length of the linker to modulate the lipophilicity of the entire conjugate. However, reducing lipophilicity and shortening the linker length decreased toxicity by limiting the uptake and delivery of cargo to mitochondria. Kulkarni et al. (2012) indicated that the substitution of electron-donating groups on the para-position of TPP phenyl rings led to a reduced electron density on the phosphorus atom, resulting in the elimination of uncoupling effects compared to the original TPP moiety, thus preventing the dissipation of mitochondrial membrane potential [50]. These modifications to the TPP structure did not impair cargo delivery to mitochondria. It is worth emphasizing that when treating neurodegenerative diseases, a mitochondrially neutral carrier is required; however, during cancer treatment, the effect of TPP on mitochondrial potential can be beneficial.

There are other compounds that act similarly to TPP, such as guanidinium, triethylammonium, pyridinium, 3-phenylsulfonylfuroxan, F16, 2,3-dimethylbenzothiazolium iodide, rhodamine 19, rhodamine 123, and DQA. Most of them are lipophilic cations, accumulating within the mitochondria of tumour cells because of the strongly negative membrane potential present in the mitochondrial matrix [184]. 

One of the most recognised lipophilic cationic fluorescent dye family known to accumulate in the mitochondria is rhodamine, including rhodamine 123, rhodamine B, and rhodamine 19 [184]. All of them are potential mitochondria-targeting moieties. Rhodamine 19 was obtained through the chemical modification of TPP moiety. Rhodamine B differs from rhodamine 19 by having two extra ethyl groups attached to the nitrogen atoms of the rhodamine structure, preventing these nitrogens from being protonated [190]. Notably, rhodamine 19, in contrast to rhodamine B, can be considered a mitochondria-targeted cationic uncoupler due to its protonophorous uncoupling effect [191]. However, probably the best studied of them is rhodamine 123. Already in 1983, Lampidis et al. observed that rhodamine 123 selectively kills carcinoma as compared to normal epithelial cells grown in vitro. What is important, at doses of rhodamine 123 which were toxic to carcinoma cells, the conversion of mitochondrial-specific to cytoplasmic-nonspecific localisation of the drug was observed before cell death [192]. Ten years later, Singer et al. showed that rhodamine 123 altered the phosphorus and glucose metabolism of the HCT-116 human colon cancer cell line. They proved that rhodamine 123 initially boosts cytoplasmic glycolysis to sustain cellular energy then it inhibits mitochondrial ATP production in HCT-116 cells [193]. 

### 5.2. Nanoparticles

Given the significant impact of cancer on human health, researchers around the world are dedicated to developing various innovative carrier systems to deliver anticancer agents to target sites, aiming to minimise harmful effects on healthy tissues. One of these approaches involves nanotechnology, with nanoparticles developed as drug delivery carriers in recent decades (summarised in [194]). Nanoparticles can specifically target various intracellular organelles, including mitochondria, lysosomes, endoplasmic reticulum, and Golgi apparatus [5]. This precise targeting can be achieved through the careful engineering of nanoparticles to focus on the desired organelle.

Nano-drug carriers for anticancer agents are significantly more effective than traditional drug delivery systems. Drug nanoparticles exhibit enhanced bioavailability, increased solubility, and the capacity to cross the blood–brain barrier, enter the pulmonary system, and can be absorbed through the tight junctions of dermalendothelial cells because of their small size and large surface area [195,196]. These nanocarrier-based systems are being utilised to treat various types of cancerous tumours [197]. Solid, colloidal particles, named nanoparticles, range in size from 10 nm to less than 1000 nm; however, less than 200 nm is the preferred size for nanomedical applications [196,198]. They offer the potential for continuous, direct, and controlled drug delivery specifically to malignant cells, enhancing drug concentration and cellular uptake. Nanoparticles can be engineered to target malignant cells accurately, ensuring precise drug delivery while avoiding interaction with normal cells. Moreover, the development of nanocarrier-based drug delivery systems has addressed several challenges, such as systemic toxicity, low oral bioavailability, poor solubility, narrow therapeutic indices, and chemoresistance [196,199,200]. The actions taken by the nanoparticles to deliver the medications to the mitochondria require a few different steps. The positively charged nanoparticles bind with the negatively charged phospholipids of the cell membrane during the first stage of intracellular absorption. The development of endolysosomes comes next. Following the breach of the endolysosomal membrane, the contents are released into the cytoplasm, and the mitochondria are targeted intracellularly [194,201]. By creating mitochondria-targeted nanocarriers that can carry medications only to the mitochondria, it is possible to get beyond a number of obstacles inside the cell and the mitochondria. It is worth mentioning that these nanocarriers help to shield the medication payloads from in vivo removal and degradation [202].

Many different nanoparticles have been engaged in anticancer therapies, including liposomes and liposome-like vehicles, polymeric and metal nanoparticles, dendrimers, quantum dots, and micelles. Some examples of their application as anticancer drugs are discussed in this review.

#### 5.2.1. Liposomes and DQAsomes

Liposomes are tiny, spherical, closed structures made of phospholipid bilayers that divide an aqueous medium from another. To provide selective delivery to the target site for in vivo application, hundreds of medications, including anticancer and antimicrobial agents, chelating agents, peptide hormones, enzymes, proteins, vaccines, and genetic materials, have been incorporated into the aqueous or lipid phases of liposomes, with varying sizes, compositions, and other characteristics (for a review see [203,204]). Despite numerous advantages, liposomes in aqueous solution often experience physical and chemical instabilities over long-term storage, primarily due to hydrolysis, oxidation of phospholipids, and aggregation. Thus, various methods, including lyophilisation, freezing, and spray drying, have been studied to stabilise liposomes [205]. 

Myocet and Doxil were the first liposome-based drugs approved for cancer treatment, both containing doxorubicin but differing in PEG-coating presence. Pharmacokinetic studies showed that free doxorubicin had a much shorter elimination half-life (0.2 hours) compared to Myocet (2.5 hours) and Doxil (55 hours), with Doxil exhibiting the longest circulation time in the blood. The particle sizes are approximately 190 nm for Myocet and 100 nm for Doxil, influencing their blood circulation times [206,207,208]. 

Another example of anticancer drug-targeting mitochondria applied in nanotechnology is lonidamine, which facilitates mtROS production and triggers programmed cell death. Liposomes loaded with lonidamine for enhanced mitochondrial targeting demonstrated the highest cytotoxic effect on A549/cDDP cells in vitro and significant antitumour activity against A549/cDDP xenografts in nude mice, compared to free lonidamine alone [209]. 

Mitochondrial targeting by nanoparticles can be achieved through active and passive approaches. Passive targeting uses the pH-dependent cationic charge of nanoparticles to bind to the negatively charged mitochondrial membrane, offering a simple and cost-effective synthesis process. However, this method can lead to NP aggregation and rapid clearance from the body. Active targeting involves functionalizing NP surfaces with lipophilic cations or mitochondrial-specific ligands, such as described above triphenylphosphonium (TPP), mitochondrial peptides, and DQAsomes [210,211]. These active targeting ligands face challenges, including immunogenicity, complex synthesis, which involves high costs, off-target toxicity, and delayed clearance from the body related with long blood circulation.

Taxol® (paclitaxel—developed in the 1980s from extracts of the leaves of the European yew tree *Taxus baccata* [212]) is used to treat ovarian, breast, non-small cell lung cancer and AIDS-related Kaposi’s sarcoma, but its low water solubility requires the use of the non-ionic surfactant Cremophor EL® for intravenous administration. Cremophor EL, however, increases toxicity and can cause hypersensitivity reactions in some patients. To address these issues, the LEP-ETU formulation of paclitaxel was developed to eliminate the need for polyoxyethylated castor oil [213]. A lyophilised liposome-based paclitaxel formulation (LEP-ETU) is sterile, stable, and easy to handle. Stability testing showed that the lyophilised LEP-ETU remained physically and chemically stable for at least 12 months when stored at 2–8°C and 25°C.

DQAsomes (liposome-like vehicles) possess a positive charge, which facilitates their accumulation in mitochondria through the electrostatic gradient, thereby inducing apoptosis. DQA is a cationic bola-amphiphile made up of two quinaldinium rings connected by 10 methylene groups, initially discovered as an antimicrobial agent. When prepared from DQA chloride, it can self-assemble into liposome-like vesicles, known as DQAsomes [214]. Utilizing DQAsomes for the targeted delivery of bioactive compounds in cancer treatment can significantly enhance their apoptosis-inducing efficacy [215]. These vehicles are able to transport both drugs and DNA into mitochondria [216]. Plasmid DNA can be delivered into mitochondria using DQAsomes through nonspecific endocytic pathways. DQAsomes induce apoptosis or necrosis by disrupting mitochondrial transmembrane potential, ATP synthesis, increased ROS production, and activation of MAPK pathways, ultimately leading to caspase-dependent apoptosis [201].

Shi et al. successfully synthesised the HER-2 peptide-PEG2000-Schiff base-cholesterol (HPSC) derivative and then incorporated it onto doxorubicin (DOX)-loaded dequalinium (DQA) chloride vesicles (HPS-DQAsomes) to treat drug-resistant breast cancer [217]. Noteworthy, the nanoparticles were designed specifically to serve as a multifunctional drug delivery system to efficiently transport therapeutical agents into the acidic tumour microenvironment (pH 6.8–5.0). The resulting HPS-DQAsomes had a particle size of about 110 nm and a spherical shape. In vitro investigation showed that HPS-DQAsomes manifested increased cytotoxicity against the DOX-resistant MCF-7/ADR cell line. Cellular uptake and mitochondria-targeting assays demonstrated that HPS-DQAsomes effectively delivered the therapeutic agent to mitochondria, inducing mitochondria-driven apoptosis. What is important is that, in in vivo assays, HPS-DQAsomes achieved favourable antitumour activity with strong apoptosis-inducing effects and no obvious systematic toxicity [217].

#### 5.2.2. Polymeric Nanoparticles

Other vehicles utilised in nanotechnology are biodegradable and biocompatible polymeric nanoparticles. Easy manufacturability and surface modification capabilities make them ideal for mitochondrial drug delivery [194,216]. They offer higher stability and more controlled payload release compared to liposomes [218]. Polymers like PLA, PGA, PLGA, and PCL can be engineered into nanoparticles that incorporate both water-soluble and insoluble payloads through processes like emulsification or nanoprecipitation; hydrophobic blocks enhance stability, while PEG increases in vivo residence time [195,203]. 

To improve selectivity, polymeric nanoparticles are modified with substances like folic acid or conjugated with antibodies targeting surface receptors that are overexpressed on specific cancer cells [219]. Poltavets et al. investigated a poly(D,L-lactide-co-glycolide) nanoparticle functionalised with folic acid and loaded with docetaxel for breast cancer treatment [220]. Docetaxel is a stronger semisynthetic derivative of paclitaxel. Since 1996, docetaxel (Taxotere®) has been a crucial chemotherapeutic drug for cancer treatment [221]. The study revealed that polymeric carriers overcame the chemopreventive effect in breast cancer cell lines overexpressing the folic acid receptor alpha (FRα). The authors showed increased cytotoxicity of docetaxel towards breast cancer cells compared to its native form, which suggests that polymeric nanocarriers enhance drug distribution and selectivity while reducing toxicity to normal cells [220].

#### 5.2.3. Metal Nanoparticles

Metal nanoparticles are particularly notable for their versatility and potential in cancer treatment. Gold nanoparticles are among the most prominent, followed by silver and magnetic nanoparticles. There are numerous research and clinical trials suggesting that metal nanoparticles can be effective in treating cancer. Additionally, using non-noble metal-based nanoparticles could provide a more cost-effective alternative to traditional chemotherapy [222]. However, noble metal nanoparticles demonstrate superior resistance to corrosion and oxidation, along with reduced cytotoxicity compared to transition metal nanoparticles [223]. 

In contrast to conventional techniques that rely on harmful chemicals as reducing and stabilizing agents in nanoparticle production, the use of biological materials such as fungi, algae, bacteria, and plant extracts offers a more sustainable alternative. This green synthesis approach not only reduces time and costs but also results in the production of non-toxic nanoparticles, making it a more environmentally friendly option [224,225]. By precisely engineering metal nanoparticles, multiple responses can be achieved under the same conditions. The accurate design of metal nanoparticles, composition and size, allows for control over their bioavailability, biological activity, and toxicity, which are major limitations of metal nanoparticles. This level of control enhances their effectiveness and safety in biomedical applications [226]. 

Gold is considered a noble element due to its non-reactivity, which makes it highly resistant to chemical oxidation, degradation, and corrosion, allowing it to maintain its structure and appearance over time [227]. Gold nanoparticles possess unique physicochemical properties that make them suitable for a wide range of biomedical applications. Research indicates that gold nanoparticles exhibit strong contrast in computed tomography (CT) imaging, facilitating the early detection of cancer. Their photothermal properties can be harnessed to administer PTT, selectively targeting and destroying cancer cells [228]. Additionally, gold nanoparticles have been employed for the identification of cancer stem cells, positioning them as a promising tool for the development of targeted therapies aimed at these cells [229].

A study by Sun and colleagues revealed that gold nanoparticles can accumulate in the mitochondria of tumour cells, disrupting their structure and function, which leads to increased oxidative stress and mitochondrial apoptosis. Additionally, gold-based nanoparticles induce metabolic stress by reducing glycolysis and depleting nutrients in tumour cells [230]. 

Currently, there has been a significant focus on utilizing gold nanoparticles for tumour-targeting strategies. Quinacrine, also referred to as mepacrine, has been utilised as an antimalarial drug for nearly a century but has recently gained attention for its potential as an anticancer agent. Current studies indicate that quinacrine may suppress cancer cell proliferation via several pathways, including modulation of autophagy, trapping of the FACT (facilitates chromatin transcription) complex, and interference with DNA repair mechanisms [231]. Satapathy et al. developed a hybrid nanoparticle composed of quinacrine and gold, which significantly inhibited cellular proliferation, induced apoptosis, and disrupted angiogenesis in vivo, leading to tumour regression in a xenograft mouse model [90]. This formulation also enhanced the production of ROS and nitric oxide in OSCC cancer stem cells, while modulating the expression of inflammatory cytokines, including IL-6, IL-1β, and TNF-α [90].

Silver nanoparticles are gaining popularity in biomedicine for applications like antimicrobial wound dressings, infection-preventing lotions, and anticancer therapies. They primarily work through mechanisms involving ROS, oxidative stress, and DNA damage [225]. In cancer cells, silver nanoparticles accumulate in mitochondria, leading to mitochondrial membrane depolarisation, ROS production, and disruption of mitochondrial stability [232,233,234,235]. Additionally, they were observed to intensify endoplasmic reticulum stress [233]. There are reports, that silver nanoparticles hold potential for promoting programmed cancer cell death and inducing apoptosis [236]. However, their anticancer effectiveness is significantly affected by factors such as size, shape, surface charge, and particularly surface coating [237].

Nanotechnology also represents the opportunity for the design of multitarget anticancer therapy. Mallik et al. developed nanoparticles targeting both the mitochondria and nucleus by combining two drugs: a mitochondria-targeting agent (α-tocopheryl succinate) and nucleus-targeting drugs (cisplatin, doxorubicin, and paclitaxel) [234]. These dual-drug nanoparticles were internalised into the acidic lysosomal compartments of HeLa cervical cancer cells via endocytosis, leading to apoptosis preceded by cell cycle arrest. They disrupted mitochondrial structure, triggering cytochrome c release, caused DNA damage, nuclear fragmentation, and disrupted the tubulin cytoskeleton.

Directly conjugating drug-targeting ligands offers simplicity and control, allowing drugs easy access to mitochondria. However, this method might decrease therapeutic effects and drug solubility. Conversely, nanocarrier systems resolve solubility issues but face challenges in optimisation due to varied compositions, potentially causing problems in mitochondrial delivery. 

## 6. Conclusions

Mitochondria play a multifaceted role that makes them essential for cancer cell survival. Their involvement in deregulated metabolism, resistance to apoptosis, enhanced autophagy, altered mitochondrial dynamics, and cancer stem cell formation significantly contribute to drug resistance and cancer progression. Consequently, targeting mitochondria has emerged as a promising therapeutic approach to overcome chemoresistance. Biologically active compounds of natural origin continue to be a leading source of new potential therapeutics. Unfortunately, many promising metabolites cannot be utilised in therapies due to their poor solubility or undesirable side effects accompanying their activity. The use of advanced platforms for compound design and ligand–receptor interaction studies enables precise modification of drug structures to enhance their physicochemical properties. Additionally, the application of nanotechnology complements classical compound modification techniques, potentially resulting in an optimal therapeutic product for targeted anticancer therapies. Modern approaches, such as lipid-based nanocarriers, nanoparticles, peptide-mediated targeting, photodynamic therapy, and immunotherapy, address challenges in anticancer drug development by enhancing precision, disrupting mitochondrial function, inducing cell death, and modulating immune responses while minimizing side effects.
